# Carbapenemase-directed therapy: optimizing antibiotic combinations against carbapenem-resistant *Klebsiella pneumoniae*

**DOI:** 10.3389/fcimb.2026.1866731

**Published:** 2026-07-07

**Authors:** Wen-bo Xu, Yi-Fei Wei, Yu-Ying Zhang, Qing-Li Guo, Lin Shi, Peng-Li Fan, Shan-Mei Wang

**Affiliations:** 1 Department of Clinical Microbiology, Henan Provincial People’s Hospital, Zhengzhou University People’s Hospital, Henan University People’s Hospital, Zhengzhou, Henan, China; 2 Department of Clinical Laboratory, Henan University People’s Hospital, Henan Provincial People’s Hospital, Zhengzhou, Henan, China; 3 Department of Pharmacology and Toxicology, College of Veterinary Medicine, Henan Agricultural University, Zhengzhou, Henan, China; 4 Department of Clinical Laboratory, Xuchang People’s Hospital, Xuchang, Henan, China; 5 Department of Pharmacy, Henan Provincial People’s Hospital, Zhengzhou, Henan, China

**Keywords:** carbapenem-resistant *Klebsiella pneumoniae*, ceftazidime-avibactam, combination susceptibility testing, KPC enzyme, NDM enzyme, polymyxin B

## Abstract

**Objective:**

Given the limited therapeutic options for infections caused by carbapenem-resistant *Klebsiella pneumoniae* (CRKP) due to multidrug resistance, this study aimed to evaluate combination therapeutic strategies based on carbapenemase profiles and resistance phenotypes.

**Methods:**

The *in vitro* activities of ceftazidime-avibactam (CZA) and polymyxin B (PMB)-based combinations was systematically evaluated against 139 CRKP isolates, including *K. pneumoniae* carbapenemase (KPC) producing strains, New Delhi metallo-β-lactamase (NDM), and KPC and NDM co-producing carbapenem-resistant *K. pneumoniae* strains (KN-CRKP). The combination effects of these antimicrobial combinations were assessed using the fractional inhibitory concentration index (FICI) and susceptibility breakpoint index (SBPI).

**Results:**

Among CZA-resistant KPC-CRKP isolates, combinations of CZA with β-lactams exhibited high synergy rates (79.7-86.5%). In PMB-resistant KPC-CRKP, PMB + CZA demonstrated substantially higher synergy rates (53.2%) than conventional PMB-carbapenem combinations (≤3.8%). Against NDM-CRKP, CZA + aztreonam (ATM) showed synergistic activity in 83.3% of isolates. Notably, in KN-CRKP, CZA + ATM produced synergistic or additive effects in all isolates, despite resistance to the individual agents. SBPI analysis further suggested substantial antimicrobial enhancement in some combinations classified as indifferent by FICI, including PMB + tigecycline (TGC).

**Conclusions:**

Carbapenemase profiles substantially influence the efficacy of combination therapies against CRKP. CZA-based combinations, particularly those combined with β-lactams or ATM, demonstrated favorable *in vitro* activity against KPC and NDM-producing CRKP, while PMB in combination with CZA partially restored activity in PMB-resistance isolates. Notably, the CZA-ATM regimen showed consistently favorable activity against both NDM-CRKP and KPC-NDM co-producing isolates. These findings support the potential value of carbapenemase profile-guided combination therapy for optimizing treatment of highly resistant CRKP infections.

## 1 Introduction

CRKP is a major multidrug-resistant (MDR) Gram-negative pathogen that poses substantial therapeutic challenges. Its emergence has been driven largely by selective pressure from widespread antibiotic application (van Duin et al., 2020). According to 2024 data from the China Antimicrobial Surveillance Network, resistance rates of *K. pneumoniae* to meropenem and imipenem were 23.4% and 22.6%, respectively (Guo et al., 2025), while polymyxin resistance reached 11.2% and continues to rise. The predominant carbapenemases in CRKP are KPC, a class A serine β-lactamase inhibited by avibactam (AVI), and NDM, a class B enzyme for which no effective clinical inhibitor is currently available (Hobson et al., 2022; Wang et al., 2022; Xu et al., 2022; Yang et al., 2023). Particularly concerning is the co-production of KN-CRKP, which represents a critical clinical challenge because of its extensive drug resistance and association with high mortality rates (Hobson et al., 2022; Rong et al., 2023). In China, ST11 remains the predominant epidemic clone among CRKP isolates and is closely associated with carbapenem resistance dissemination, multidrug resistance, and adverse clinical outcomes, further complicating infection control and treatment strategies (Yang et al., 2022).

CZA is effective against KPC-CRKP but inactive against metallo-β-lactamase-producing strains, including those carrying NDM (Zasowski et al., 2015). the last-r PMB is considered esort therapeutic option for infections caused by multi-drug resistant and extensively drug-resistant Gram-negative organisms (Rodríguez-Santiago et al., 2021). Nevertheless, the expanding clinical use of β-lactams and PMB has promoted the emergence of resistant isolates, further limiting available therapies and posing a substantial public health threat (Hu et al., 2024). Recent clinical studies have further demonstrated that infections caused by CZA-resistant CRKP are associated with poor clinical outcomes and complex resistance mechanisms, highlighting the increasing therapeutic challenges posed by emerging CZA resistance (Wu et al., 2026). Particularly alarming is the increasing prevalence of PMB-resistant CRKP, which is frequently associated with pan-drug resistance (PDR) (Rezk et al., 2026). Together, these challenges underscore the urgent need for effective combination strategies.

Despite growing interest in combination therapy, the available evidence remains limited and fragmented. Recent *in vitro* studies have suggested that CZA plus meropenem (MEM) exhibits relatively strong *in vitro* activity among tested combinations against KPC-CRKP (Liu et al., 2025), whereas CZA plus aztreonam (ATM) is currently considered one of the most biologically rational and better-supported combination strategies for NDM or other MBL-producing isolates (Liu et al., 2022; Bawankar et al., 2026). However, most published studies have examined heterogeneous CRKP collections, have not specifically enriched for CZA-resistant, PMB-resistant, or dual-resistant isolates, and have provided only limited data on KN-CRKP. This gap is clinically important because KN-CRKP has been increasingly reported internationally and is associated with an extensively drug-resistant phenotype (Haller et al., 2019; Shahid et al., 2022). Moreover, although recent multicenter real-world studies have suggested possible clinical advantages of CZA-based regimens over PMB-based regimens for CRKP infections, PMB remains widely used in settings where access to newer agents is limited or resistance to frontline β-lactam/β-lactamase inhibitor regimens has already emerged (Chen et al., 2022; Zhuang et al., 2025). Therefore, a systematic, carbapenemase-guided comparison of CZA and PMB-based combinations against highly resistant CRKP subgroups is still lacking.

Against this background, we designed the present study to evaluate the *in vitro* activity of CZA and PMB-based combination regimens against CRKP stratified by carbapenemase genotype, including KPC-CRKP, NDM-CRKP, and KN-CRKP, with particular emphasis on CZA- and PMB-based combinations and isolates co-producing multiple carbapenemases. By specifically enriching for isolates resistant to CZA, PMB, or both, and by integrating FICI and susceptibility breakpoint index (SBPI) analyses (Milne and Gould, 2010), we aimed to define genotype-specific interaction patterns, identify combinations capable of restoring activity in highly resistant subpopulations, and provide experimental evidence to support carbapenemase-guided optimization of combination therapy.

## 2 Materials and methods

### 2.1 Materials

A total of 139 CRKP isolates were collected from individual patients at Henan Provincial People’s Hospital between January 1 and December 31, 2023. To construct a cohort of highly resistant isolates, strains resistant to PMB (MIC ≥ 2 mg/L), CZA (MIC ≥ 16 mg/L) (Institutional 2024 IIC, 2024), or both were preferentially included. Based on carbapenemase genotype confirmation, 115 KPC-producing CRKP (KPC-CRKP), 11 NDM-producing CRKP (NDM-CRKP), and 13 KN-CRKP) isolates were selected for combination antimicrobial susceptibility testing. The quality control isolates used in this study were Escherichia coli ATCC 25922 and K. pneumoniae ATCC 700603, obtained from the National Institute for the Control of Pharmaceutical and Biological Products (China).

This study received approval from the Ethics Committee of Henan Provincial People’s Hospital, which waived the requirement for informed consent because no identifiable patient information was disclosed.

### 2.2 Bacterial identification

Initial screening of isolates was performed using the BD PHOENIX™ M50 automated identification system (BD, USA). Species-level confirmation as *K. pneumoniae* was conducted using the MALDI Biotyper^®^ system (Bruker Daltonik GmbH, Bremen, Germany).

### 2.3 Confirmation of CRKP

CRKP isolates were confirmed by routine laboratory methods. Antimicrobial susceptibility testing was interpreted according to the Clinical and Laboratory Standards Institute (CLSI) guidelines (M100, 34th edition, 2024) (Institutional 2024 IIC, 2024) and U.S. Food and Drug Administration (FDA) breakpoints, where applicable. Isolates were defined as CRKP if they were resistant to at least one carbapenem agent (imipenem, meropenem, or ertapenem).

#### 2.3.1 Detection of carbapenemase types

Carbapenemase genotyping was conducted using the NG-Test^®^ CARBA5 lateral flow assay (NG Biotech, Guipry, France), which detects the five most prevalent carbapenemase families (KPC, NDM, VIM, IMP, and OXA-48-like). NG-Test CARBA 5 has been validated in previous studies as a reliable alternative to PCR for clinical epidemiological investigations (Jenkins et al., 2020).

### 2.4 Antimicrobial susceptibility testing

Selection of antimicrobial combinations was guided by the Infectious Diseases Society of America (IDSA) recommendations for drug-resistant Gram-negative infections, expert consensus on combination susceptibility testing for carbapenem-resistant Gram-negative bacteria (Chemotherapy EBoCJoIa, 2023; Tamma et al., 2024), and preliminary in-house screening results. CZA-based regimens included combinations with meropenem, imipenem, amikacin, or aztreonam, whereas PMB-based regimens included combinations with meropenem, imipenem, CZA, and TGC (Tyers and Wright, 2019; Liu et al., 2021). Checkerboard microbroth dilution assays were performed in accordance with CLSI guidelines (M07, 12th edition) (Institutional 2024 IIC, 2024). Given the large number of clinical isolates and antimicrobial combinations evaluated, independent biological replicates were not performed for each isolate–combination pair. Instead, technical replicates were conducted to ensure the consistency of MIC, FICI, and SBPI determinations. Quality control strains (*E. coli* ATCC 25922 and *K. pneumoniae* ATCC 700603) were included in each experimental batch to ensure methodological consistency.

### 2.5 Calculation and interpretation of FICI and SBPI

Drug combination effects were evaluated using FICI, based on IDSA guidance and expert consensus (Tamma et al., 2024). FICI values of ≤ 0.5, > 0.5 to ≤ 1, >1 to ≤2, and >2 were interpreted as indicating synergy, additivity, indifference, and antagonism, respectively. The SBPI was calculated as follows: SBPI = (Susceptible breakpoint of drug A/MIC of drug A in combination) + (Susceptible breakpoint of drug B/MIC of drug B in combination). Higher SBPI values generally indicate greater enhancement of *in vitro* antimicrobial activity following combination treatment (Milne and Gould, 2010). Because no universally accepted interpretive criteria or absolute cutoff values have been established for SBPI, this metric was used in the present study as a relative comparative indicator for evaluating differences among antimicrobial combinations rather than as an independent determinant of synergistic activity.

### 2.6 Statistical analysis

Data analysis was performed using SPSS version 27.0 (IBM Corp., Armonk, NY, USA). Categorical variables were summarized as counts and percentages (n [%]) and compared using Fisher’s exact test. Continuous variables were analyzed using one-way analysis of variance (ANOVA) or the Kruskal-Wallis test, as appropriate. Statistical analyses of synergy rates and SBPI distributions were performed at the isolate population level across different antimicrobial combination regimens. P values < 0.05 were considered statistically significant.

## 3 Results

### 3.1 Resistance rates of CRKP to seven antimicrobial agents

**Figure 1:**
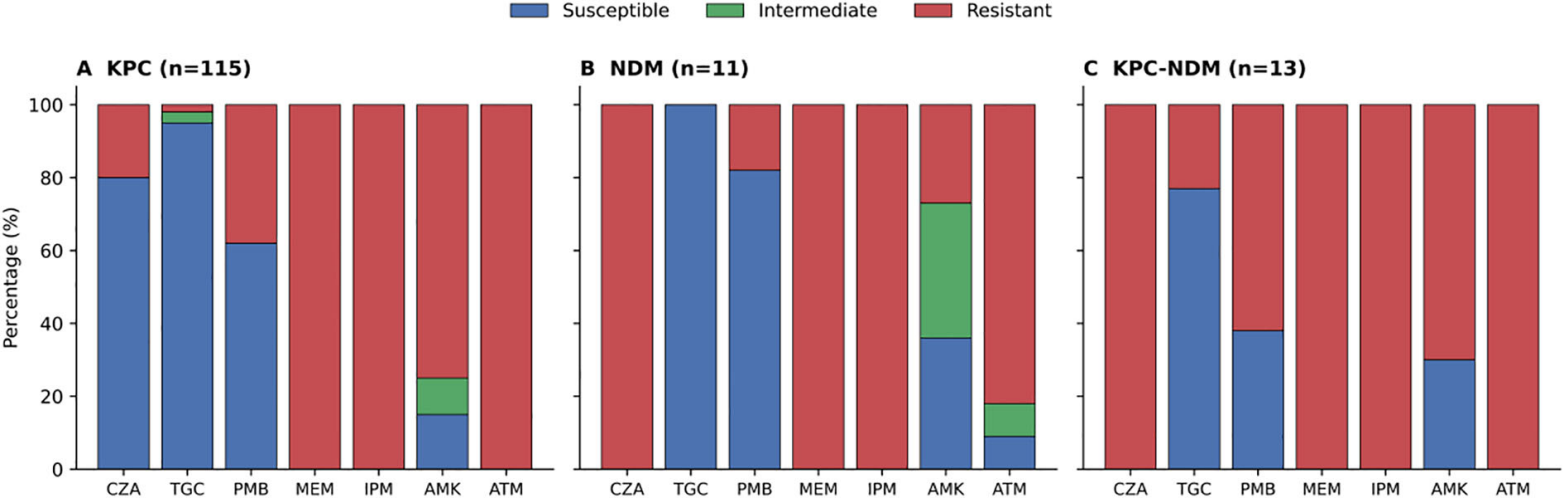
Results of antibiotic susceptibility testing of 139 CRKP isolates to seven AMAs A. **(A-C)** Antimicrobial susceptibility profiles of KPC-producing, NDM-producing, and KPC-NDM-producing CRKP. Abbreviations: CZA, ceftazidime-avibactam; PMB, polymyxin B; IPM, imipenem; ATM, aztreonam; TGC, tigecycline; MEM, meropenem; AMK, amikacin.

Antimicrobial susceptibility profiles varied substantially among 139 CRKP isolates according to carbapenemase genotype (**Figure 1**). Among KPC-CRKP isolates (n = 115), TGC and CZA exhibited the greatest *in vitro* activity, with susceptibility rates of approximately 96% and 80%, respectively, followed by PMB (approximately 62%). In contrast, resistance to MEM, IPM, and ATM was universal, while AMK showed only limited activity, with approximately 16% of isolates classified as susceptible and 10% as intermediate. Among NDM-CRKP isolates (n = 11), TGC retained complete activity (100% susceptibility), whereas PMB and AMK demonstrated moderate activity, with susceptibility rates of approximately 82% and 36%, respectively; an additional 36% of isolates showed intermediate susceptibility to AMK. By comparison, all NDM-CRKP isolates were resistant to CZA, MEM, and IPM, and most were also resistant to ATM (approximately 82%). KN-CRKP isolates (n = 13) exhibited the broadest resistance phenotype: TGC was the only agent that retained substantial activity (approximately 77% susceptibility), whereas susceptibility to PMB and AMK decreased to approximately 38% and 31%, respectively (**Figure 1**), and all isolates were resistant to CZA, MEM, IPM, and ATM. Collectively, these findings identify TGC as the most consistently active agent across all carbapenemase genotypes, supporting its continued role as an important salvage option for CRKP infections. In contrast, MEM, IPM, and ATM showed minimal to no *in vitro* activity. Notably, reduced susceptibility to PMB was observed across all genotype groups (**Figure 1**), suggesting that polymyxin-resistant CRKP is already present in Henan Province, China, and may represent an emerging challenge for hospital infection control.

### 3.2 in vitro antibacterial activity of drug combinations against KPC-CRKP

#### 3.2.1 Activity of CZA-based combinations

Among all KPC-CRKP isolates, the *in vitro* activity of CZA-based combinations differed significantly (*P* < 0.001). Combinations of CZA with MEM, ATM, or IPM showed the highest synergy rates (86.2%, 86.5%, and 79.7%, respectively) and produced at least 4-fold reductions in MIC_90_ relative to monotherapy (**Supplementary Table 1**). These synergy rates were significantly higher than those of CZA combined with TGC or AMK (P < 0.05). In addition, the CZA-AMK combination had the lowest mean SBPI, which differed significantly from all other combinations (P < 0.001) (**Table 1**).

**Table 1:** Comparison of synergistic results of CZA *in vitro* combined with antibacterial drugs on KPC-CRKP.

Combination Groups	No. of isolates by FICI (N, %)				SBPI			
	Synergistic (≤0.5)	Additive (>0.5 to 1)	Indifferent (>1 to 2)	Antagonistic (>2)	Range	Mean	Median	P
CZA + MEM(n=29)	25(86.2) ^a^	1(3.4)	3(10.4) ^b^	0	0.375-18	16.25	18	<0.001
CZA								
MEM								
CZA + ATM(n=74)	64(86.5) ^a^	1(1.4)	9(12.2) ^b^	0	0.375-18	16.60	18	
CZA								
ATM								
CZA + IPM(n=74)	59(79.7) ^a^	9(12.1)	6(8.1) ^b^	0	0.375-18	17.33	18	
CZA								
IPM								
CZA + TGC(n=29)	7(24.1)	6(21.0)	15(51.7)	1(3.4)	1.25-20	18.25	20	
CZA								
TGC								
CZA + AMK(n=84)	2(2.4)	15(17.9)	66(78.6)	1(1.2)	0.28-16.5	7.10^c^	4.5	
CZA								
AMK								

^a,b^The synergistic and indifferent effects of CZA + MEM, CZA + ATM, and CZA + IPM are statistically significantly different from those of other combination groups (P < 0.05).

^c^The mean SBPI of the CZA + AMK group is significantly lower than that of all other groups (P < 0.001).

**Figure 2:**
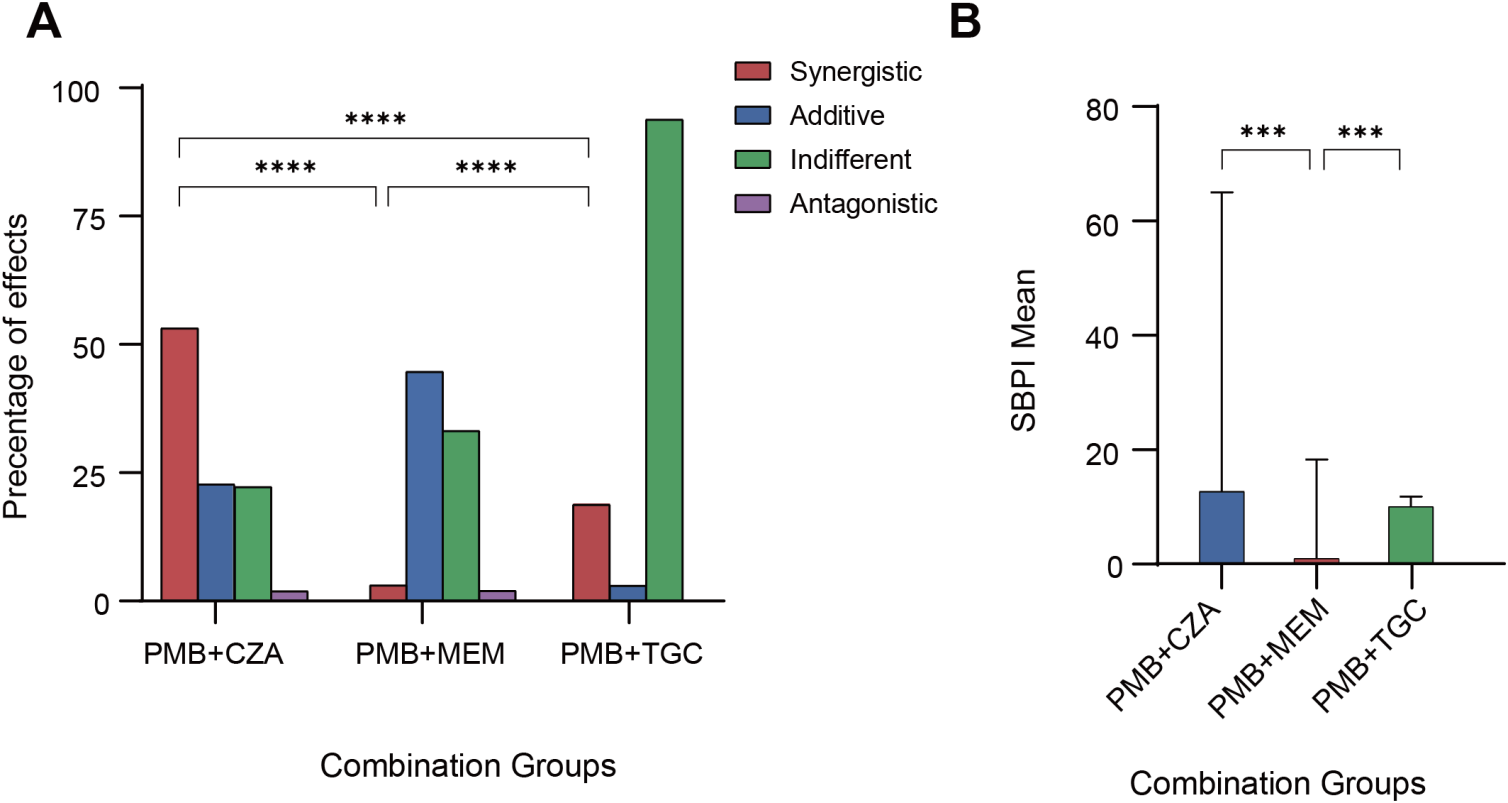
Comparison of the *in vitro* synergistic activity of CZA-based combinations against CZA-resistance KPC-CRKP isolates. **(A)** Distribution of combination effects among CZA-Resistant KPC-CRKP isolates. **(B)** SBPI values for CZA-based combinations against CZA-Resistant KPC-CRKP. Statistical significance was calculated using one-way ANOVA with either Dunnett correction or Tukey correction for multiple comparison: *P < 0.05, **P < 0.01, ***P < 0.001, ****P < 0.0001; ns, not significant.

Among CZA-resistant KPC-CRKP isolates, significant differences in activity among CZA-based combinations remained evident (P < 0.05). Synergy rates for CZA + MEM, CZA + ATM, and CZA + IPM were 87.5%, 50.0%, and 75.0%, respectively, compared with 25.0% for both CZA + TGC and CZA + AMK. Furthermore, the proportion of additive effects was significantly higher in the CZA + TGC group than in the other combination groups (**Figure 2A**). Subsequent SBPI analysis revealed that the average SBPI was highest for CZA + TGC (mean 18.25), followed by CZA + IPM (mean 17.33), CZA + ATM (mean 16.60), and CZA + MEM (mean 16.25). In contrast, the lowest value was recorded for CZA + AMK (mean 7.10, **Table 1**). Notably, despite its lower synergy rate, the CZA + TGC group showed the highest mean SBPI (18.03), suggesting enhanced bactericidal activity in a subset of isolates (**Figure 2B**).

Taken together, the FICI and SBPI results indicate that CZA in combination with MEM, ATM, or IPM provides comparatively greater *in vitro* activity relative to combinations involving TGC or AMK. CZA + MEM, CZA + ATM, and CZA + IPM exhibited high synergy rates without evidence of antagonism, highlighting their potential *in vitro* activity against KPC-CRKP isolates. By contrast, CZA + TGC demonstrated a relatively low synergy rate yet the highest SBPI, suggesting that it may still provide a meaningful enhancement in antimicrobial activity. CZA + AMK displayed the poorest overall activity, indicating relatively limited potential utility against KPC-CRKP isolates.

#### 3.2.2 Activity of PMB-based combinations

The *in vitro* activities of PMB-based combinations against KPC-CRKP differed substantially. Among the three regimens evaluated, PMB + CZA exhibited the highest synergy rate, with synergistic activity observed in 22.6% (26/115) of isolates, which was significantly higher than those of the other combination groups (P < 0.05). By comparison, synergy rates were 3.8% (3/78) for PMB + MEM and 8.0% (9/113) for PMB + TGC (**Table 2**). Antagonism was uncommon across all regimens, occurring in 3.5%, 0%, and 2.7% of isolates, respectively, whereas indifferent interactions predominated, accounting for 59.1%-68.1% of isolates. The mean SBPI of PMB + CZA (18.37) also exceeded that of PMB + TGC (11.64) and PMB + MEM (5.76), indicating superior *in vitro* activity (**Table 2**). Consistent with the FICI results, PMB + CZA also produced the greatest reductions in MIC_90_, lowering the MIC_90_ of PMB from 8 to 1 mg/L and that of CZA from 32 to 2 mg/L (**Supplementary Table 2**). PMB + TGC likewise reduced the MIC_90_ values of both agents (PMB, 8 to 1 mg/L; TGC, 2 to 0.5 mg/L) (**Supplementary Table 2**). In contrast, PMB + MEM showed only limited improvement in MIC values, with no reduction in the MIC_90_ of PMB and only a ≥ 2 decrease in the MIC_90_ of MEM (128 to 64 mg/L). Furthermore, mean SBPI values followed the order PMB + CZA (18.37) > PMB + TGC (11.64) > PMB + MEM (5.76), with the PMB + CZA group showing the highest mean SBPI (P < 0.001).

**Table 2:** Comparison of synergistic results of PMB in vitro combined with antibacterial drugs on KPC-CRKP. .

Combination Groups	No. of isolates by FICI (N, %)				SBPI			
	Synergistic (≤0.5)	Additive (>0.5 to 1)	Indifferent(>1 to 2)	Antagonistic (>2)	Range	Mean	Median	P
PMB + CZA(n=115)	26(22.6) ^a^	17(14.8)	68(59.1)	4(3.5)	1.25-68	18.37	36	<0.001
PMB								
CZA								
PMB + MEM(n=78)	3(3.8)	22(28.2)	53(67.9)	0	0.26-16.02	5.76^b^	8	
PMB								
MEM								
PMB + TGC(n=113)	9(8.0)	24(21.2)	77(68.1)	3(2.7)	3-24	11.64	12	
PMB								
TGC								

^a^the synergistic effects of PMB + CZA are statistically significantly different from those of other combination groups (P < 0.05).

^b^the mean SBPI of the PMB + MEM group is significantly lower than that of all other groups (P < 0.001).

**Figure 3:**
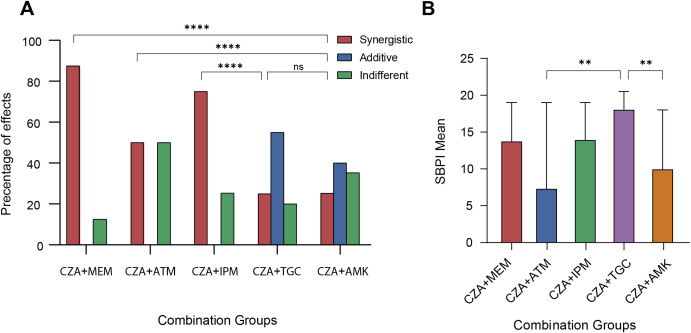
Comparison of the *in vitro* synergistic activity of PMB-based combinations against PMB-Resistance KPC-CRKP isolates. **(A)** Combined effects of PMB-Resistant KPC-CRKP. **(B)** SBPI of PMB-Resistant KPC-CRKP. Statistical significance was calculated using one-way ANOVA with either Dunnett correction or Tukey correction for multiple comparison: *P < 0.05, **P < 0.01, ***P < 0.001, ****P < 0.0001; ns, not significant.

Among PMB-resistant KPC-CRKP isolates (**Figure 3**), significant intergroup differences persisted (P < 0.001). PMB + CZA also achieved the highest synergy rate (53.2%), significantly surpassing PMB + TGC (19.1%) and PMB + MEM (3.1%) (P < 0.05, **Figure 3A**). PMB + CZA showed a higher mean SBPI (12.78) than PMB + TGC (9.89); however, this difference was not statistically significant (**Figure 3B**). Notably, the PMB + MEM group exhibited the lowest mean SBPI among all three combination groups, with a value significantly lower than those observed for the other regimens (P < 0.001).

#### 3.2.3 Synergistic activity of both CZA-based and PMB-based combinations against dual-resistant KPC-CRKP

**Figure 4:**
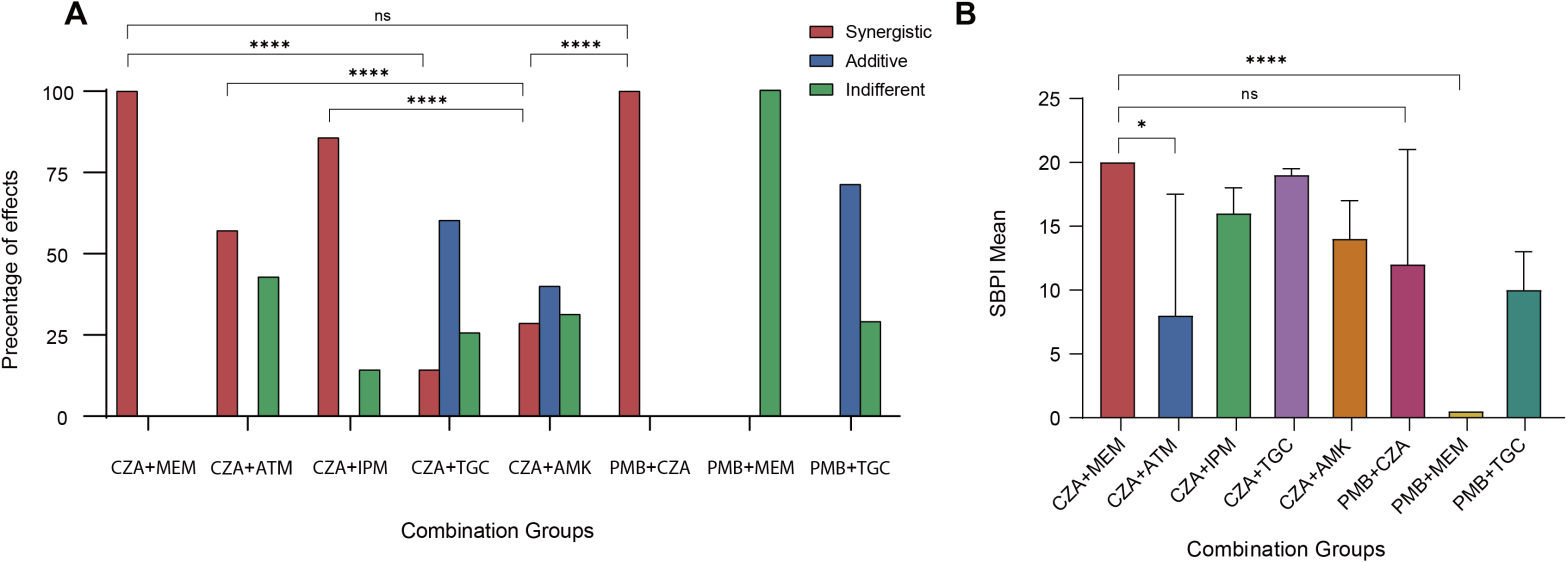
*In vitro* synergistic activity of CZA- and PMB-based combinations against CZA and PMB Dual-resistant KPC-CRKP. **(A)** Distribution of combination effects of CZA- and PMB-Based Regimens Against Dual-resistant KPC-CRKP. **(B)** SBPI of CZA and PMB Dual-resistant KPC-CRKP. Statistical significance was calculated using one-way ANOVA with either Dunnett correction or Tukey correction for multiple comparison: *P < 0.05, **P < 0.01, ***P < 0.001, ****P < 0.0001; ns, not significant.

The *in vitro* activities of CZA and PMB-based combinations differed markedly among the CZA and PMB-dual-resistant KPC-CRKP isolates, the synergistic effects of CZA + MEM and PMB + CZA are especially statistically significantly different from those of CZA + TGC, PMB + MEM, and PMB + TGC (P < 0.05, **Figure 4A**). In the FICI-based analysis (**Figure 3A**), CZA + MEM and PMB + CZA exhibited the most favorable interaction profiles, with synergistic effects observed in all tested isolates. CZA + IPM also retained a high synergy rate (85.7%), whereas CZA + ATM remained synergistic in 57.1% of isolates. By contrast, the activities of CZA + TGC and CZA + AMK were more heterogeneous: only 14.3% and 28.6% of isolates, respectively, showed synergy, while the remaining isolates were classified predominantly as additive or indifferent. Among the PMB-based regimens, PMB + MEM showed no synergistic activity and was uniformly indifferent, whereas PMB + TGC was characterized mainly by additive effects (71.4%) with the remainder being indifferent. Notably, no antagonistic interactions were observed in this dual-resistant subset (**Figure 4A**).

The SBPI analysis further discriminated the relative *in vitro* activity of the tested combinations (**Figure 4B**). CZA + MEM and PMB + CZA exhibited significantly higher synergy rates than other combinations (P < 0.05, **Figure 4A**), and showed the highest mean SBPI values (19.2, 19.8, respectively). Pairwise comparisons indicated that the mean SBPI of CZA + MEM was higher than that of CZA + ATM (P < 0.05) and markedly higher than that of PMB + MEM (P < 0.001), which showed the lowest mean SBPI by approaching zero, while no significant difference was observed between CZA + MEM and PMB + CZA. Importantly, although CZA + TGC showed only limited FICI-defined synergy, it yielded an SBPI comparable to that of CZA + MEM, suggesting that its antibacterial enhancement in this subset may be more closely associated with substantial MIC reduction than by attainment of the FICI synergy threshold.

### 3.3 Antibacterial activity of antibiotic combinations against NDM-CRKP

Among the CZA-based combinations, CZA + ATM exhibited the most favorable interaction profile, with synergistic activity observed in 5 of 6 tested isolates (83.3%) and additive activity in the remaining isolate (16.7%); no indifferent or antagonistic interactions were identified (**Table 3**) This regimen also showed consistently high SBPI values, with a median of 18.0 and a narrow range of 16.5–18.0. In contrast, CZA + MEM was uniformly indifferent in all 4 tested isolates (100.0%), with a fixed SBPI of 8.25. CZA + AMK displayed modest activity, with synergistic and additive effects each observed in 1 of 6 isolates (16.7%), whereas 4 of 6 isolates (66.7%) remained indifferent; the median SBPI was 16.5 (range, 8.5–16.5). Notably, CZA + TGC did not produce FICI-defined synergy in any of the 4 tested isolates, but yielded additive activity in 1 isolate (25.0%) and indifferent activity in 3 isolates (75.0%), with uniformly high SBPI values of 20.0. Among the PMB-based regimens, PMB + CZA showed the highest numerical synergy rate, with synergistic activity observed in 2 of 11 tested isolates (18.2%) and additive activity in 1 isolate (9.1%). This combination also exhibited the widest SBPI distribution, ranging from 0.5 to 72.0, with a median of 20.0, indicating marked isolate-to-isolate heterogeneity. PMB + TGC and PMB + MEM were predominantly indifferent (81.8% and 80.0%, respectively). PMB + TGC yielded one synergistic and one additive interaction, whereas PMB + MEM showed no synergistic interactions and a lower median SBPI of 8.0 (range, 0.3125–12.0).

**Table 3:** Results of the in vitro combined antibiotics susceptibility test of NDM-CRKP.

Number^a^	CZA (KB (mm))	PMB MIC/(mg/L)	CZA + MEM		CZA + ATM		CZA + TGC			CZA + AMK			PMB + MEM			PMB + CZA			PMB + TGC	
			FICI	SBPI	FICI	SBPI	FICI	SBPI	FICI		SBPI	FICI		SBPI	FICI		SBPI	FICI		SBPI
77	15	≤0.5	/	/	/	/	/	/	/		/	2		12	0.5		72	2		24
210	6	4	/	/	/	/	/	/	/		/	1.02		4.5	1.02		16.5	0.63		12
22	17	≤0.5	/	/	0.08	18	/	/	1.02		16.5	1		12	1.02		20	1.13		12
75	14	≤0.5	/	/	/	/	/	/	/		/	2		12	2		68	1.25		16
173	15	≤0.5	2	8.25	0.08	18	1	20	1		16.5	1		8	1		20	1.5		12
42	6	≤0.5	/	/	0.08	18	/	/	0.09		8.5	/		/	0.27		20	0.38		12
116	17	≤0.5	2	8.25	0.08	18	1.02	20	1.02		16.5	1.02		8	1.02		20	2		12
189	16	8	/	/	/	/	/	/	/		/	2		0.3125	2		0.5	1.1		12
115	10	≤0.5	2	8.25	0.52	16.5	1.02	20	1.02		16.5	1.02		8	1.02		20	2		12
82	6	1	2	8.25	0.08	18	1.01	20	1.01		16.5	1.01		8	1.01		20	1.5		12
78	8	≤0.5	/	/	/	/	/	/	/		/	2		12	8		65	2		16
n	11	11	4	4	6	6	3	4	6		6	10		10	11		11	11		11

^a^Sample number from the 11 NDM-CRKP isolates.

/, No combined susceptibility test results available. nf, The number of actually detected strain.

whereas the remaining combinations generally exhibited limited activity and were predominantly characterized by indifferent interactions, whereas most other combinations, particularly PMB-based regimens -were dominated by indifferent interactions. An additional finding was the partial discordance between FICI- and SBPI-based assessments: although PMB + TGC and, to a lesser extent, CZA + AMK rarely met the FICI threshold for synergy, their SBPI values suggested that breakpoint-related antimicrobial enhancement may still have occurred in selected isolates.

### 3.4 Antibacterial activity of antibiotic combinations against KN-CRKP

Among the 13 KN-CRKP isolates, distinct activity patterns were observed among the tested antimicrobial combinations against KN-CRKP (**Table 4**). Among the CZA-based combinations, CZA + ATM showed the most favorable activity profile, with the highest synergy rate (81.8%, 9/11) and additive effects in the remaining 2 isolates (18.2%), while no indifferent or antagonistic interactions were identified. The corresponding SBPI values were also consistently high, with a median of 18.0 and a range of 1.25-18.0. By contrast, CZA + AMK showed uniformly indifferent interactions in all 11 tested isolates (100.0%), despite a wide SBPI distribution (range, 0.28-17.5; median, 0.28). CZA + IPM showed limited activity, with synergy observed in 2 of 10 isolates (20.0%), additivity in 1 isolate (10.0%), and indifference in the remaining 7 isolates (70.0%); SBPI values ranged from 0.375 to 18.0.

**Table 4:** Results of the *in vitro* combined antibiotics susceptibility test of KN-CRKP.

Number^a^	CZA (KB (mm))	PMB (MIC/(mg/L))	CZA + MEM		CZA + ATM		CZA + TGC		CZA + AMK		CZA + IPM		PMB + MEM		PMB + CZA		PMB + TGC	
			FICI	SBPI	FICI	SBPI	FICI	SBPI	FICI	SBPI	FICI	SBPI	FICI	SBPI	FICI	SBPI	FICI	SBPI
60	17	>2	/	/	/	/	/	/	/	/	/	/	2	0.26	2	0.3125	0.3125	20
81	17	1	/	/	/	/	/	/	/	/	/	/	4	9	2	36	1.25	24
118	15	1	2	8.125	0.08	18	1.02	16.25	1.02	16.5	/	/	1.02	8	1.02	20	1.03	12
6	15	≤0.5	/	/	0.75	1.25	/	/	2	0.28	2	0.375	1	16	1.02	20	2	12
15	14	≤0.5	/	/	0.08	18	/	/	2	0.28	2	0.375	1.02	8	1.02	20	1.5	12
103	17	>2	/	/	0.08	18	/	/	2	0.28	2	0.375	/	/	2	0.5	/	/
107	14	>2	/	/	0.08	18	/	/	2	0.28	2	0.375	/	/	2	0.5	/	/
109	6	>2	/	/	0.08	18	/	/	1.02	16.5	2	0.375	/	/	2	0.5	/	/
73	6	≤0.5	/	/	0.08	18	/	/	1.02	17.5	2	0.375	/	/	1.02	20	2	12
98	6	>16	/	/	0.08	18	/	/	2	0.28	2	0.375	/	/	2	0.5	/	/
46	15	16	/	/	0.08	18	/	/	2	0.28	0.13	17	/	/	0.63	2.5	1.06	12
47	15	8	/	/	0.08	18	/	/	2	0.28	0.14	/	/	/	0.5	2	0.56	8
48	21	16	/	/	0.56	18	/	/	1.5	16.5	0.56	18	/	/	1.06	12	1.06	12
n	13	13	1	1	11	11	1	1	11	11	10	9	5	5	13	13	9	9

^a^Sample number from the 13 KN-CRKP isolates./, No combined susceptibility test results available. n, The number of actually detected strains.

For PMB-based regimens, PMB + CZA was tested most frequently, but its activity was largely indifferent, with 11 of 13 isolates (84.6%) classified as indifferent, 1 isolate (7.7%) as additive, and only 1 isolate (7.7%) as synergistic. Its SBPI values were highly heterogeneous, ranging from 0.3125 to 36.0, with a median of 2.5. PMB + TGC also showed predominantly indifferent interactions (77.8%, 7/9), with 1 additive interaction (11.1%, 1/9) and 1 additive (11.1%, 1/9) result; however, its SBPI values remained consistently moderate to high (range, 8.0-24.0; median, 12.0). PMB + MEM showed the least favorable overall profile among the PMB-based regimens: no synergistic interactions were observed in the 5 tested isolates, while 3 isolates (60.0%) showed indifference, 1 (20.0%) showed additivity, and 1 (20.0%) showed antagonism.

Overall, CZA + ATM demonstrated the most consistent *in vitro* activity against KN-CRKP in this dataset and was the only combination to achieve synergistic or additive effects in all tested isolates. By contrast, most other regimens particularly CZA + MEM and PMB + MEM, were dominated by indifferent interactions. The discordance between FICI and SBPI observed for CZA + TGC, CZA + AMK, and PMB + CZA suggests that some regimens may substantially reduce combination MICs without consistently meeting the conventional FICI threshold for synergy.

### 3.5 Clinical data of combinations against KN-CRKP

Clinical outcomes for the 13 patients with KN-CRKP infection are summarized in **Supplementary Table 3**. Notably, only 2 patients (15.38%) received CZA + ATM therapy and 2 patients (15.38%) received CZA + PMB therapy, whereas 9 patients (69.23%) received heterogeneous alternative regimens. Meanwhile, bacterial replacement occurred in 0% (0/2), 50.0% (1/2), and 22.2% (2/9) of cases in the respective groups. Persistent non-clearance was observed exclusively in the “other” regimen group, affecting 33.3% (3/9) of patients. However, given the limited sample size and substantial treatment heterogeneity, these observations should be interpreted cautiously and are presented primarily as exploratory clinical findings that may provide preliminary support for future investigation of combination strategies against KN-CRKP.

## 4 Discussion

Therapeutic options for CRKP infections remain severely limited due to the widespread prevalence of multidrug resistance. Combination therapy has therefore emerged as a promising strategy to enhance antimicrobial efficacy and extend the therapeutic utility of existing agents (Tyers and Wright, 2019). Notably, the marked heterogeneity of carbapenemase enzymes among CRKP isolates necessitates the development of mechanism-based combination strategies (Liu et al., 2021). However, systematic comparative analyses evaluating antimicrobial combinations across distinct resistance mechanisms remain scarce. In this context, the present study integrated *in vitro* susceptibility profiles with combination activity analyses across multiple carbapenemase genotypes, thereby providing a more comprehensive framework for mechanism-guided therapeutic optimization.

Consistent with both our dataset and accumulating evidence, CZA-based combinations exhibited genotype-dependent activity, with the most pronounced effects observed in KPC-producing CRKP (Wang et al., 2020; Wu et al., 2024). Specifically, combinations of CZA with β-lactam agents-including meropenem, imipenem, and aztreonam demonstrated higher synergy rates and greater MIC_90_ reductions compared with non-β-lactam partners such as tigecycline and amikacin. Notably, prior studies have identified CZA plus MEM as one of the most active *in vitro* combination regimens, while recent evidence further indicates that CZA in combination with ATM, MEM, or imipenem (IPM) displays robust synergistic activity across different KPC variants. Importantly, our findings further suggest that even CZA-resistant KPC-CRKP isolates may partially regain susceptibility when treated with CZA-β-lactam combinations, suggesting partial restoration of β-lactam activity mediated by avibactam. Previous studies have shown that rapid replacement and evolution of blaKPC variants in ST11 CRKP can contribute to the emergence of CZA resistance during infection, highlighting the dynamic adaptive potential of KPC-producing isolates under antimicrobial pressure (Yang et al., 2025). Collectively, these findings support the potential utility of β-lactam-based combinations against KPC-producing CRKP isolates.

In contrast to the consistent performance of CZA-based regimens, PMB-based combinations exhibited substantial variability and overall limited efficacy, particularly in resistant subpopulations (Liu et al., 2024). Specifically, the PMB -MEM combination showed poor synergistic activity against PMB-resistant KPC-CRKP isolates, likely due to high baseline resistance to both agents (Diep et al., 2017; Gaibani et al., 2019). However, the PMB-CZA combination demonstrated a markedly improved synergy profile, especially in PMB-resistant isolates, where it achieved the highest synergy rate and superior SBPI values among PMB-based regimens (Wang et al., 2022). This observation may be related to complementary antibacterial mechanisms, in which PMB disrupts the outer membrane while CZA inhibits cell wall synthesis, thereby enhancing drug penetration and suppressing resistant subpopulations. Supporting this concept, a recent study demonstrated that exogenous glutathione could partially reverse meropenem resistance in CRKP through modulation of membrane permeability and bacterial metabolic pathways, highlighting the importance of permeability-associated resistance reversal strategies in highly resistant isolates (Yi et al., 2023). Notably, in isolates co-resistant to both PMB and CZA, PMB-CZA maintained activity comparable to CZA-β-lactam combinations, underscoring its potential as an alternative combination strategy against highly resistant KPC-CRKP isolates.

For MBL-producing CRKP, particularly NDM-positive isolates, our results demonstrate a distinct combination activity pattern characterized by selective synergy of CZA-ATM. Although CZA monotherapy is intrinsically ineffective against MBL producers, the addition of ATM restored antimicrobial activity, yielding high synergy rates and consistently elevated SBPI values. These findings are consistent with current clinical recommendations and reinforce the mechanistic rationale that ATM remains stable to MBL hydrolysis while avibactam inhibits co-produced serine β-lactamases (Guzek et al., 2024; Wang et al., 2025). However, these findings should be interpreted with caution. The number of NDM-producing isolates included in this study was relatively limited, and certain antimicrobial combinations were evaluated in only a small subset of isolates. Therefore, although CZA-ATM demonstrated the most favorable activity profile within the NDM-CRKP subgroup, the observed differences among treatment regimens should be considered preliminary and require validation in larger collections of NDM-producing CRKP isolates.

Of particular concern is the emergence of KPC-NDM co-producing CRKP (KN-CRKP), which exhibited the broadest resistance spectrum and the fewest available therapeutic options in our dataset. In this context, CZA-ATM was the only regimen that consistently exhibited synergistic or additive effects across all tested isolates, even in cases with dual resistance to both agents (Gao et al., 2020; Rong et al., 2023). In contrast, PMB-based combinations -including PMB-CZA and PMB-TGC-were predominantly classified as indifferent despite occasional MIC reductions. Preliminary clinical observations from the limited cohort were broadly consistent with the *in vitro* findings; however, because of the small sample size and multiple confounding factors, these observations should be considered exploratory only and are insufficient for reliable assessment of comparative clinical efficacy. Taken together, these findings suggest that CZA-ATM exhibited comparatively favorable *in vitro* activity against KN-CRKP isolates and highlight the importance of enzyme profile-guided combination selection in this high-risk subgroup (Li et al., 2024).

Beyond regimen-specific findings, this study also provides methodological insight through the integration of FICI and SBPI for combination evaluation. While FICI remains the standard metric for defining synergy, our results demonstrate that it may incompletely reflect antibacterial enhancement in certain combinations, particularly those associated with substantial MIC reductions but not meeting strict synergy thresholds. In contrast, SBPI, despite lacking standardized interpretive criteria, captured additional patterns of antimicrobial enhancement and revealed discordance in several regimens (e.g., PMB-TGC and CZA-TGC), suggesting that breakpoint-based metrics may complement conventional synergy assessments. In the present study, SBPI was primarily used as a relative comparative indicator rather than an absolute measure of synergistic activity. Therefore, the combined use of FICI and SBPI may provide a more comprehensive framework for evaluating antimicrobial combinations (Milne and Gould, 2010; Tian et al., 2021).

Interestingly, several combinations, particularly CZA+TGC, demonstrated notable discordance between FICI- and SBPI-based assessments. Although CZA + TGC exhibited relatively low rates of FICI-defined synergy, it consistently produced high SBPI values and substantial MIC reductions in selected isolates. These findings suggest that FICI and SBPI may reflect different dimensions of combination activity, with FICI primarily evaluating interaction patterns and SBPI more closely representing the extent of susceptibility restoration (Milne and Gould, 2010; Okoliegbe et al., 2021a). Previous studies have similarly indicated that checkerboard-based FICI analyses may underestimate antibacterial enhancement observed in dynamic pharmacodynamic assays, including time-kill experiments (White et al., 1996). Therefore, certain combinations that fail to meet conventional FICI-defined synergy thresholds may still achieve potentially meaningful reductions in combination MICs. In this context, SBPI may provide complementary information beyond conventional interaction-based assessments and help identify combinations with potential therapeutic value that could otherwise be overlooked using FICI alone (Okoliegbe et al., 2021b). Further validation using dynamic pharmacodynamic models is warranted.

Taken together, these findings delineate distinct, mechanism-specific combination patterns across CRKP subtypes. CZA-β-lactam combinations demonstrated favorable activity against KPC-producing CRKP, whereas PMB-CZA may represent a potential salvage option for polymyxin-resistant isolates. In addition, CZA-ATM consistently exhibited the most consistent *in vitro* activity against MBL-producing and KPC-NDM co-producing CRKP isolates in our dataset. Collectively, these findings underscore the importance of enzyme profile-guided combination strategies for the management of highly resistant CRKP infections.

This study has several limitations. First, the present work was based primarily on *in vitro* checkerboard assays and did not incorporate dynamic pharmacokinetic/pharmacodynamic (PK/PD) simulations or *in vivo* infection models. Therefore, the observed combination activities may not fully reflect antimicrobial activity under clinically relevant conditions under clinically relevant conditions. Future studies integrating dynamic pharmacodynamic models, animal infection experiments, and clinical validation will be important to further evaluate the translational potential of these regimens. Second, the checkerboard method provides a static assessment of antimicrobial interactions and cannot fully capture dynamic bacterial responses during antimicrobial exposure. In addition, although technical replicate experiments were performed, independent biological replicates for each isolate-combination pair were not systematically conducted, which may limit assessment of interexperimental variability. Third, resistance mechanisms were characterized primarily by carbapenemase genotyping, while broader genomic determinants potentially associated with antimicrobial resistance or heteroresistance were not comprehensively investigated. Future whole-genome sequencing and molecular analyses may help clarify additional mechanisms influencing combination activity. Finally, the clinical observations derived from the KN-CRKP cohort involved a limited sample size and heterogeneous treatment regimens, which introduced multiple potential confounding factors. Nevertheless, as an exploratory study specifically enriched for highly resistant CRKP subpopulations, the present work still provides preliminary experimental evidence supporting carbapenemase-guided combination screening and may serve as a useful foundation for future mechanistic and translational investigations.

## 5 Conclusions

In conclusion, dual-index evaluation based on FICI and SBPI provides additional insights into the assessment of antimicrobial combination activity in highly resistant CRKP subpopulations, including isolates resistant to PMB, resistant to CZA, and those co-producing multiple carbapenemases. CZA-based β-lactams combinations exhibited relatively high synergistic activity against CZA-resistant KPC-CRKP, while PMB + CZA demonstrated comparatively greater *in vitro* activity than conventional PMB-based combinations in PMB-resistant isolates. Moreover, CZA + ATM demonstrated consistently favorable *in vitro* activity against NDM- and KN-CRKP isolates. The observation that several combinations restored *in vitro* activity despite resistance to the individual agents further highlights the value of carbapenemase profile-guided therapeutic strategies. Overall, these findings highlight the potential value of combination susceptibility testing for the management of extensively drug-resistant CRKP infections.

## Data Availability

The original contributions presented in the study are included in the article/**Supplementary Material**. Further inquiries can be directed to the corresponding author.
