# Supplementary material for: Carbapenemase-directed therapy: optimizing antibiotic combinations against carbapenem-resistant Klebsiella pneumoniae
Source: Front Cell Infect Microbiol. 2026 Jul 7;16:1866731. doi: 10.3389/fcimb.2026.1866731 (PMC13384889; doi:10.3389/fcimb.2026.1866731)
Supplement: Supplementary file 5 [file Table5.docx]

**Table S3. Preliminary clinical observations of antimicrobial regimens in patients with KN-CRKP infection**

These data are presented as preliminary exploratory observations only. Interpretation is limited by the small sample size, heterogeneous treatment regimens, and potential clinical confounding factors. Therefore, no definitive conclusions regarding comparative clinical efficacy can be drawn from these findings.
